# Corneal approach 20 Guage vitrectomy system for the management of congenital cataract

**DOI:** 10.12669/pjms.322.9149

**Published:** 2016

**Authors:** Mariya Nazish Memon, Sadia Bukhari, Israr Ahmed Bhutto

**Affiliations:** 1 Dr. Mariya Nazish Memon, FCPS, FICO(Australia). Department of Peadiatric Ophthalmology &Strabismus, Al-Ibrahim Eye Hospital/Isra Post Graduate Institute of Ophthalmology, Karachi, Pakistan; 2 Dr. Sadia Bukhari, FCPS. Department of Peadiatric Ophthalmology &Strabismus, Al-Ibrahim Eye Hospital/Isra Post Graduate Institute of Ophthalmology, Karachi, Pakistan; 3 Dr. Israr Ahmed Bhutto, FCPS, FICO(USA). Department of Peadiatric Ophthalmology &Strabismus, Al-Ibrahim Eye Hospital/Isra Post Graduate Institute of Ophthalmology, Karachi, Pakistan

**Keywords:** 20 Gauge vitrectomy, Congenital cataract, Corneal approach, Cataract surgery

## Abstract

**Objective:**

To evaluate the efficacy and complications of 20 gauge vitrectomy via corneal approach for the management of congenital cataract.

**Method:**

We performed anterior capsular vitreorhexis, lens matter aspiration (LMA), primary posterior vitrectorhexis and anterior vitrectomy via corneal approach using 20 gauge vitrectomy system in children younger than two years of age with congenital cataract between January 2014 to December 2014. The intra and postoperative complications were observed.

**Results:**

Twenty nine eyes of 21 children were included in this study. Congenital cataract surgery using 20 gauge vitrectomy system via corneal approach did not reveal any intra operative complication. Post operatively all children were able to freely open their operated eyes. Conjunctival congestion at the incision site in four eyes and mild anterior chamber reaction in 8 eyes were seen on 1^st^ daywhich resolved at one week follow up. Other major post operative complications such as inflammatory membrane, irregular pupil, posterior/anterior syneache and opacification of visual axis were not seen during follow up period.

**Conclusion:**

The 20-gauge vitrectomy system via corneal approach is easy to perform, is less time consuming, safe and effective for the management of congenital cataract in younger children.

## INTRODUCTION

Congenital cataracts are responsible for about 10% of all childhood blindness worldwide.^1^ Early surgical intervention and visual rehabilitation is necessary to prevent the development of cataract induced sensory deprivation amblyopic. The routine surgical procedure for congenital cataract is to perform continuous curvilinear capsulorhexis (CCC), aspirate the soft lens matter, perform posterior circular capsulorhexis and remove the anterior vitreous.^2^, ^3^ Although several steps of adult cataract surgery can be adopted during pediatric cataract surgery but Pediatric eye is different from adult eye. Thin and elastic sclera with positive vitreous pressure in pediatric eyes can lead to repeated shallowing of anterior chamber during surgery. Elasticity of anterior capsule and soft, sticky lens matter makes the capsulorhexis and cataract removal more time consuming and difficult.^4^, ^5^ After better understanding of complexity of pediatric eye and advances in microsurgical techniques, Surgery via corneal approach under close chamber technique with vitrectomy cutter has now become popular for the management of congenital cataract in young children.^6^-^8^ “Vitrectorhexis” is the term used when anterior capsular opening is made with the help of vitrectomy cutter, it is best alternative to continuous curvilinear capsulorhexis in young children because of elasticity of capsule and high chances for capsulorhexis to extend.^9^-^11^ Small corneal incisions are easily closed with stromal hydration or with single suture. Limited intervention and shorter duration of surgery reduces the chances of intra ocular inflammation and improves the comfort level post operatively.

The aim of current study was to evaluate the efficacy, intra and post operative complications of 20 gauge vitrectomy via corneal approach for the management of congenital cataract.

## METHODS

This study was conducted in the Department of Pediatric Ophthalmology and Strabismus, Al-Ibrahim Eye Hospital, Karachi from January 2014 to December 2014. Approval was obtained from ethical review committee of the Hospital. Twenty nine eyes of 21 children younger than two years of age presented with congenital cataract were included in this study. It was a convenience sampling interventional study. Patient with history of glaucoma, uveitis, and with other anterior segment abnormalities such as anterior segment dysgenesis, aniridia were excluded. After written informed consent from the parents or guardian, all patients underwent detailed ophthalmic examination including visual acuity, intra ocular pressure, slit lamp examination for the anterior segment and dilated fundus examination. Ocular ultrasound was performed in children with no fundus view to rule out posterior segment pathology.

All Surgeries were performed under general anaesthesia (GA) by a single surgeon after fully dilatation of pupil. After standard aseptic measures, two corneal side ports were made at 2 and 10’ O clock with 20 gauge micro vireo retinal (MVR) blade to introduce automated vitrector-aspirator probe and self retaining anterior chamber maintainer (ACM) cannula (lewicky-visitec international) for irrigation. Instruments were tight fit at the incision site to prevent leakage during surgery and stabilize the depth of anterior chamber. Laureate (ALCON, Switzerland) machine with Irrigation/Aspiration (I/A)cut mode was used to perform surgery with the cutting rate of 250-300cpm (cuts per minute) and maximum suction pressure was 450mmHg. Anterior and posterior capsular opening achieved with Vitrectorhexis. Cutting edge of vitrector probe faced down in contact with anterior capsule to make initial central capsular opening and then cutter faced up to enlarge the size of anterior capsulotomy. Lens matter was aspirated with the help of same probe. Cutter and ACM cannula sites were exchanged where needed. After cataract aspiration, posterior capsulotomy and anterior vitrectomy was done with the same vitrector prob. The size of posterior capsular opening was little bit smaller than anterior capsulorhexis. Both corneal side ports were closed with single suture of 10/0 vicryl. Subconjunctival injection of gentamycin and dexamethasone given at the end of surgery. Topical antibiotic and steroid were given post-operatively and tapered off during 4-6 weeks time.

Stability of anterior chamber (AC), wound leak and rate of iris prolapse were observed intra operatively. Children were followed up on 1^st^ day and 1^st^ week and then 1, 3 and 6 months post operatively. Hand held slit lamp was used to examine the child and Chloral hydrate syrup (25mg/kg) was given orally for sedation if the child was not cooperative for complete eye examination. Patients were looked for any post operative complications i-e conjunctival hyperaemia at the site of incision, corneal oedema, anterior chamber reaction, pupil shape (regular/irregular), anterior or posterior syneache and posterior capsular opacification on every post operative visit. Data were analyzed by SPSS 20. Frequencies and percentages were calculated for categorical variables like intra and post operative complications. Mean ± SD were computed for age and duration of follow up post operatively. Aphakic glasses were prescribed to children on 1^st^ week post op visit.

## RESULT

Twenty nine eyes of 21 children with congenital cataract were included in this study (8 girls and 13 boys). Thirteen children had unilateral cataract and 8 children had bilateral (Table-I). The mean age at the time of surgery was 12.8±3.9 months and duration of follow-up was 11±5.2 months (3-20 months).

**Table-I:** Demographic characteristics (N=29).

	Frequency(%)
*Age(months)*	
Mean	12.8m+/-3.9m
Mean Range	7-18m
*Gender*	
Male	13(62%)
Female	08(38%)
*Laterality*	
Unilateral	13(62%)
Bilateral	08(38%)
*Follow-up period(months)*	
Mean	11m+/-5.2m
Range	3-20m

Intra operatively anterior chamber was stable in all the patients and iris prolapse were not seen. On 1^st^ day post op most of the children were able to freely open their operated eyes. Conjunctival congestion at the incision site in four eyes and mild anterior chamber reaction in 8 eyes was observed on 1^st^ day which resolved at one week post operatively (Table-II). Other major post operative complications such as inflammatory membrane, irregular pupil, posterior/anterior syneache and opacification of visual axis were not seen during whole follow up period.

**Table-II:** Post operative complications.

	1^st^ post op day	1 week post op
*Conjunctival congestion*		
Yes	04eyes (14%)	
No	25eyes (86%)	29eyes (100%)
*Anterior chamber reaction*		
Yes	08eyes(27.5%)	
No	21eyes(72.5%)	29eyes (100%)

## DISCUSSION

Infancy and early childhood is an important time for visual development. The eyes grow and emmetropise, Vision improves, Stereopsis matures and Accommodation develops. If child has congenital cataract then cataract surgery and visual rehabilitation should be done as soon as possible to provide adequate visual stimulus and promote visual development.

The routine surgical approach for pediatric cataract is to perform anterior CCC with cystotome needle, lens matter aspiration with I/A Simco cannula, posterior CCC with cystotome needle and anterior vitrectomy or using pars plana approach, posterior capsule incised with 20G MVR blade and anterior vitreous removed with 20G vitrectomy system.^4^ This method has some disadvantages. There are high chances of extension of capsulorhexis towards periphery because of capsule elasticity. Removal of lens matter with Simco I/A cannula require comparatively bigger incision of about 2mm or more which is less self sealed. Fluid out flow during surgery can lead to instability of anterior chamber, repeated iris prolapsed, iris depigmentation and post operative intra ocular inflammation. Eyes of the younger children are not fully mature so the judgment of anatomical location of pars plana is not very precise^11^, pars plana approach for posterior capsulotomy and anterior vitrectomy can lead to surgical trauma and posterior segment complications such as hemorrhage, cystoids macular oedema, retinal tear and retinal detachment. At the end of the surgery incision need to be closed with 10/0 nylon sutures which need removal under general anesthesia, another risk and inconvenience to children and their families.

The main aim of paediatric ophthalmologist is to reduce surgical trauma during cataract surgery in young eyes and reduce the risk of intra and post operative complications and this aim can be achieved by adopting minimally invasive close chamber vitrectomy technique via corneal approach for the management of congenital cataract. With 20 G vitrectomy system, corneal incision size is 0.9mm which matches with the size of vitrectomy cutter and irrigation ACM cannula so no wound leak and iris prolapsed and anterior chamber is stable during surgery. On the other hand, clear corneal incision avoid intra operative bleeding which is usually seen in cases of limbal and scleral incision. Anterior and posterior vitrectorhexis are more precise, controlled and quick with vitrectomy cutter and incisions in clear cornea reduces the risk of posterior segment complication.^6^-^8^

Wilson Jr compared the Vitrectorhexis and manual CCC in post-mortem children eyes after enucleation (age range four days-16 years) and observed that Vitrectorhexis is the better option in children younger than five years while manual CCC is for children of five years or older.^12^ Another study also compared the same and concluded that Vitrectorhexis is the best option in children younger than six years as compare to manual CCC which is quite appropriate for children of six years or older age group.^13^ Ozgur Ilhan and coworkers also concluded that Vitrectorhexis is safe and easy technique in children younger than six years of age.^14^

Lots of Literature available for pars planacapsulotomy and anterior vitrectomy using different gauges (G) i-e 20G,23G,25G^6^, ^7^, ^15^ but it is not so about close chamber vitrectomy technique via corneal approach for the management of congenital cataract. Suyan Li and coworkers evaluated the efficacy and complications of 23G vitrectomy via corneal approach for the treatment of pediatric cataract and observed stable anterior chamber intraoperatively. Most of the children could blink normally on 1^st^ post operative day. Only mild post operative inflammation observed which disappeared up to one week post op. Other complications such as corneal oedema, inflammatory exudates/membrane in AC, bleeding, low or high intra ocular pressure (IOP) and posterior capsular opacification (PCO) were not seen during follow up period.^8^

Haung YM and coworkers evaluated the safety and efficacy of two surgical techniques:23G trans corneal vitrectomy system (group A) and phacoemulsification I/A system with anterior vitrectomy (Group B)for the management of congenital cataract in children and observed that AC was stable and no iris prolapsed was in group A during surgery while in group B 58% of children showed iris prolapsed because of wound leak and anterior chamber instability during procedure and concluded that 23G trans corneal limbal vitrectomy is safe and effective than phacoemusification I/A in pediatric cataract surgery.^16^

In present study, 20G vitrectomy system was used through clear corneal incisions for the management of congenital cataract and we did not come across any intra operative complication. Incision was tight fit, AC was stable and no iris prolapsed. Post operatively conjunctival congestion at incision site in 4 eyes and mild anterior chamber reaction in 8 eyes were observed on 1^st^ post operative day which subsided by one week post operatively. Other post operative complication such as inflammatory membrane in AC, irregular pupil, anterior or posterior syneache and visual axis opacification were not seen during whole follow up period. Another advantage in present study was use of 10/0 vicryl to close corneal wound which absorb spontaneously within 6-8 weeks. This is to avoid risk of GA and inconvenience to the children and their parents.

Although surgical technique in present study has several advantages but study itself has few limitations i-e small sample size and limited follow up. Further studies with large sample size and longer follow up are recommended with the control group comparing the efficacy and complications between traditional method and close chamber micro surgical technique.

## CONCLUSION

The 20-gauge vitrectomy system via corneal approach is easy to perform, is less time consuming, safe and effective for the management of congenital cataract in younger children.
